# Evidence that non-cognate proteinogenic amino acids generate immunogenic neoepitopes

**DOI:** 10.1016/j.jbc.2026.111349

**Published:** 2026-03-06

**Authors:** Kenneth J. Rodgers

**Affiliations:** School of Life Sciences, The University of Technology, Sydney, Australia

**Keywords:** non-protein amino acid, neoepitope, mistranslation, autoimmunity

## Abstract

Most organisms rely on 20 DNA-encoded canonical amino acids (AAs) for protein synthesis. However, hundreds of non-canonical amino acids (NCAAs) occur in nature, many of which are plant secondary metabolites. Some NCAAs have been identified as proteinogenic and can mimic canonical AAs in mammalian protein synthesis. The tRNA synthetases responsible for AA recognition have evolved to discriminate against other canonical AAs, but they can activate NCAAs that share close structural similarity with a canonical AA. Some of these proteinogenic NCAAs play a role in plant chemical warfare (allelopathy). When incorporated into proteins, they lead to the production of high levels of non-native proteins, which can negatively impact the health of competing plants or predators. Although the impact of proteinogenic NCAAs on human health is not fully understood, it has generally been attributed to the accumulation of non-native, misfolded proteins in cells, similar to the mechanism of plant allelopathy. More recently, however, the ability of proteinogenic NCAAs to generate immunogenic neoepitopes has been demonstrated *in vivo*. In this review, we summarize emerging experimental evidence supporting NCAA-induced immune responses as a mechanism of NCAA toxicity in humans and its potential as a therapeutic approach for certain cancers.

## General introduction to amino acids

**Figure 1:**
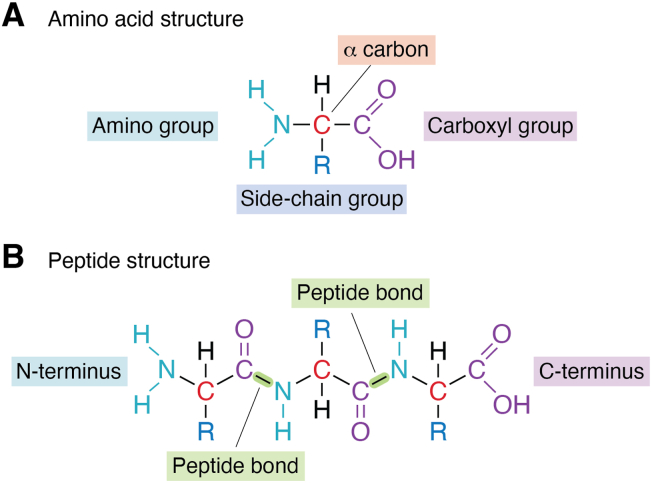
**Amino acid and protein structure**. *A*, amino acids consist of a central carbon atom (α-carbon) which is bonded to a carboxyl group, an amino group, a hydrogen atom, and a unique side-chain group (R). *B*, proteins polymerise by forming peptide bonds through a condensation reaction in which the OH group from the carboxyl terminus on one amino acid and the H group from the amino terminus of a second amino acid combine to form a water molecule.

Amino acids (AAs) are thought to have arisen through chance chemical reactions within a primitive milieu (1). Their importance lies in their ability to form polymers and generate functional molecules. It is likely that, during the early stages of Darwinian evolution, the canonical AAs were selected from the limited AA pool available at that time (2, 3). This selection might also have been influenced by thermal, chemical, and photochemical stability (2, 4). Peptide bonds between AAs readily form through a condensation reaction, allowing them to polymerize in a head-to-tail fashion, since each new AA added produces a new terminus with an identical functional group (Fig. 1). The stability of the peptides generated could also have played a role in the canonical AA selection process, with the less stable peptides being discarded.

The canonical (or protein) AAs are organic compounds consisting of an α-carbon bonded to a carboxyl group, an amino group, a hydrogen atom and a unique side chain group, making them α-AAs (Fig. 1*A*). The diversity of the side-chain group confers a range of functions on the proteins. Once incorporated into proteins, AAs can undergo post-translational modifications, further increasing the functional capabilities of the protein (5, 6). In addition to being building blocks for peptides and proteins, the 20 DNA-encoded canonical AAs play essential roles in a range of metabolic pathways in most organisms (7). Chirality of AAs is based on the molecular configuration around the α-carbon atom (Fig. 1*A*). The canonical AAs are all L-AAs. D-amino acids, in which the spatial arrangement around the α-carbon is the mirror image of the L-form, are also found in nature. The biological homochirality of proteins is thought to increase their stability; however, the reason for the selection of the L-isomer remains speculative.

## Plant amino acids

The genetic code comprises 22 amino acids, the 20 canonical amino acids found in all organisms, plus selenocysteine and pyrolysine, which are encoded only in some genomes (8). Plants produce a wide variety of non-canonical AAs (NCAAs) not encoded in protein synthesis. NCAAs, such as ornithine, citrulline, and homoserine, are metabolic intermediates in the central metabolism of plants and are present in most plant species (9). Other NCAAs are secondary metabolites not involved in central metabolism; they typically have a more sporadic distribution across the plant kingdom and often provide protection against pests and pathogens. The synthesis of secondary NCAAs is often regulated in response to environmental triggers (10). For example, 5-hydroxynorvaline can accumulate in plant leaves in response to insect herbivory (10). Canavanine, a secondary metabolite found only in a limited number of plants (11), is a structural analogue of arginine and exerts its toxicity by interfering with arginine-related metabolism, including nitric oxide synthase activity and the incorporation of arginine into proteins (9). L-canavanine is also a major nitrogen storage compound in the seeds of many plants (12) and can be used in energy-yielding respiratory reactions as well as recycling of nitrogen (13). NCAAs also include post-translationally or chemically modified forms of canonical AAs.

Plants are exposed to D-amino acids produced by microorganisms in the rhizosphere (the zone surrounding the roots), which often have an inhibitory effect on plant growth (14). Plants, however, can produce D-amino acids through the action of racemases, and these molecules can have important signaling functions (15). β-AAs, in which the amino group is attached to the β-carbon (the next carbon atom in the chain) rather than the adjacent α-carbon (Fig. 1), are present in many plant species (Kudo *et al*., 2014). Some β-amino acids, such as β-tyrosine, have defensive functions in plants (Yan *et al*., 2015), but can also be essential components of primary metabolism (9).

Generally, the most toxic plant AAs are those with the ability to mimic canonical AAs in protein synthesis. This subgroup of NCAAs is known as proteinogenic NCAAs and is exploited by plants to wage chemical warfare against predators or to inhibit the growth and survival of competing plants (16, 17). The extent to which proteinogenic NCAAs affect human health remains poorly understood. Most *in vitro* and *in vivo* studies have focused on the toxicity associated with the deposition of aberrant NCAA-containing proteins in cells and tissues, with long-lived or post-mitotic cells being particularly vulnerable (18). More recently, it has been demonstrated at the organismal level that proteinogenic NCAAs can trigger an immune response due to the neoantigens produced by their presence in proteins (19, 20). It should be noted that the majority of NCAAs are not proteinogenic and can affect human and animal health through mechanisms other than incorporation into proteins (reviewed in (21)).

## Translational errors in protein synthesis

**Figure 2:**
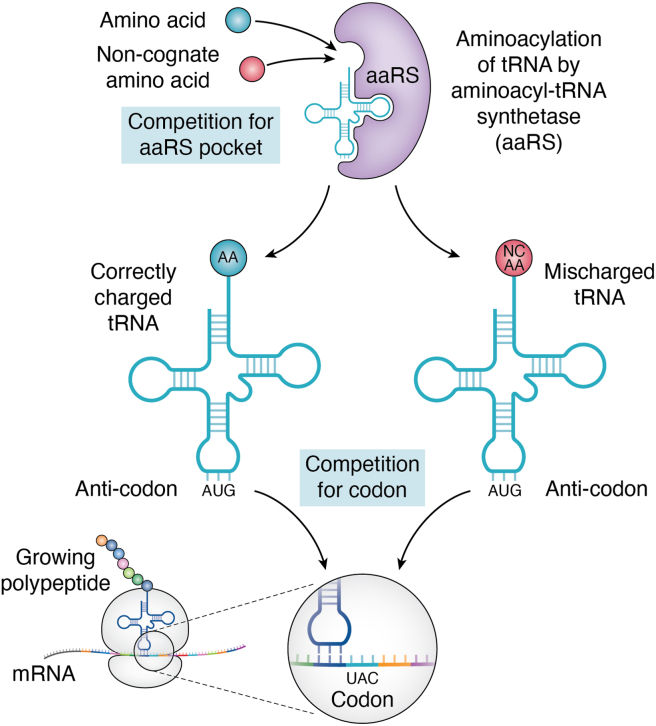
**Schematic representation of tRNA mischarging with a non-canonical amino acid (NCAA)**. A proteinogenic NCAA can compete with a cognate amino acid for tRNA charging (aminoacylation) by the aminoacyl tRNA synthetase, and if not removed by a proofreading mechanism, can compete with the correctly charged tRNA for insertion into a protein.

The translation of genetic information into functional proteins involves three distinct steps, each employing different strategies to prevent or limit errors. The highest error rates occur during translation, where mistakes commonly result from mischarging, in which there is an error in the aminoacylation of tRNAs by aminoacyl-tRNA synthetases (aaRS) (22) (Fig. 2). The typical mistranslation rate is around one in 10^4^ incorrect AAs in a growing protein chain, increasing to as high as one in 10^3^ incorrect AAs under cell stress (23). Since AAs are small molecules, the limited number of contact points between the substrate and the synthetase makes discrimination difficult (24, 25). Quality control systems are adapted to minimize naturally occurring errors involving cognate AAs however, the translational machinery can still be subverted by NCAAs (24).

Cognate AAs are attached to their respective tRNAs by aaRSs, and the ability of these enzymes to distinguish between cognate and non-cognate substrates is a major determinant of the fidelity of the genetic code (24) (Fig. 2). For an NCAA to be incorporated into a protein during translation, it must first be recognized by an aaRS. The tRNA is then aminoacylated by the aaRS in a two-step reaction. The first step involves ATP-dependent activation of the NCAA, followed by ligation of the NCAA to the tRNA through an aminoacyl ester bond (8). For approximately half of the aaRSs, the specificity of this process for cognate *versus* non-cognate AAs is about 3000 to 1, which approximates the overall error rate for protein synthesis (26), and no further quality control is required (8). However, the remaining aaRSs have ‘near-cognate’ substrates and rely on editing mechanisms to proofread non-cognate canonical amino acids. aaRS editing can occur either pre-transfer or post-transfer, depending on whether products of the first or second step of the aminoacylation reaction are hydrolyzed (8). For example, phenylalanyl-tRNA synthetase (PheRS) requires editing to prevent the delivery of Phe-tRNA^Tyr^ to the ribosome and protect against mistranslation of phenylalanine (Phe) codons as tyrosine (Tyr) (8). The proteinogenic NCAA azetidine-2-carboxylic acid (A2C) is an analogue of the canonical amino acid proline. A2C can be misacylated onto Pro-tRNA and Ala-tRNA. Both of the tRNA synthetases possess editing systems; however, misacylated Ala-tRNA^A2C^ is hydrolyzed, whereas Pro-tRNA^A2C^ can evade these mechanisms, resulting in mistranslated proteins containing A2C in place of proline (27).

## Amino acid mimicry in protein synthesis

When the toxicity of NCAAs was first systematically investigated in the 1960s, many were found to have growth-inhibitory effects on bacteria, due to their mistaken incorporation into proteins (16, 28). Proteinogenic NCAAs could have been among the earliest toxins, as they can disrupt the growth of even the most primitive organisms reliant on protein synthesis for survival. For example, fescue grasses release *meta*-tyrosine into the soil, which replaces Phe during protein synthesis (28), thereby inhibiting the growth of competing plants (29). Proteinogenic NCAAs retained within an organism can also deter grazers, herbivores, or pathogens (29). The evolution of more selective tRNA synthetases represents a host defense strategy against autotoxicity. For example, the jack bean (*Canavalia ensiformis*) has evolved an arginyl-tRNA synthetase capable of preventing canavanine insertion into its own proteins (30). In many cases, tRNA-synthetases that have evolved a higher selectivity for the canonical AA than a proteinogenic NCAA mimic are known as “advanced” synthetases. Predators can also adopt this strategy; the bruchid beetle possesses an advanced arginyl-tRNA synthetase with low affinity for canavanine, reducing its susceptibility to canavanine-induced toxicity (31). *In vivo* exposure to NCAAs, which typically have a much lower affinity for the tRNA-synthetase than the parent amino acid, generally results in only a modest increase in the synthesis of non-native proteins. Levels of competing canonical AAs are therefore a key factor determining the extent of non-native protein synthesis. Dedicated quality control systems in the cytosol, nucleus, and organelles help maintain proteome integrity by recognizing and removing damaged proteins (32, 33). However, while most cells and tissues can tolerate a modest increase in non-native protein production due to well-developed defense systems and an ability to distribute non-degradable proteins among daughter cells, post-mitotic cells are more vulnerable (34).

## Characteristics of proteomimetic non-canonical amino acids

Since mimicry in protein synthesis occurs only for NCAAs that closely resemble a canonical AA in size, shape, and charge, larger canonical AAs are more likely to have proteinogenic mimics that meet these criteria (18, 35). Oxidized forms of aromatic amino acids such as *ortho*-tyrosine, *meta*-tyrosine, and 3,4-dihydroxyphenylalanine (DOPA) are proteinogenic, since the addition of an oxygen molecule to the aromatic ring has minimal impact on size, shape, and charge (36, 37). The mistaken incorporation of NCAAs into newly synthesized proteins is a random process, in which the NCAAs and the cognate AA compete for a specific aaRS (35). Within a protein, each residue has an equal chance of being replaced by an NCAA. However, substitution of solvent-exposed (surface) residues could have less impact on the protein’s tertiary structure compared to the replacement of an internal (buried) residue (38). The chemistry of an NCAA can alter the folding, stability, and function of a protein, and both residue-specific and site-specific replacement of canonical amino acids with NCAAs is widely used in protein engineering (39).

## Neoepitope generation by non-cognate amino acids

It was first recognized in the 1960s that peptides generated from mutated proteins synthesized by tumors, known as neoantigens, can be recognized as non-self and stimulate an immune response (40). This forms part of the immune surveillance system against malignancy, in which major histocompatibility complex (MHC) molecules present a sample of the tumor proteome on the cell surface for inspection by T cells (41). MHC class I proteins display peptides generated by proteasomal degradation of cytosolic proteins, whereas MHC class II proteins present peptides derived from endosomal or lysosomal proteolysis (41). Neoantigen peptides presented on MHC molecules to CD8^+^ T cells (MHC I) or CD4^+^ T cells (MHC II) (42) that induce immunity are referred to as neoepitopes. Only a small proportion of neoantigens create neoepitopes (43). Since neoantigens are foreign to the immune system, neoepitope-specific T cells are not subject to central tolerance (44).

**Figure 3:**
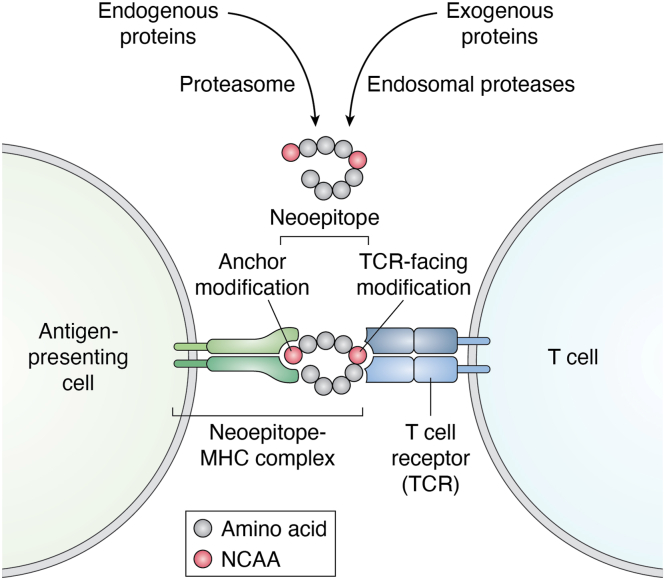
**Mechanisms increasing the antigenicity of a neoepitope due to the presence of an NCAA**. Neoepitopes can be generated from endogenous and exogenous proteins by the action of proteases. The presence of an NCAA residue in the neoepitope can create an anchor that increases the binding affinity of the peptide towards the MHC molecule. An NCAA residue facing the T-cell receptor (TCR) binding site can increase the TCR-binding properties of the neoepitope, thereby enabling recognition by T-cells.

Most cancer mutations are non-synonymous single-nucleotide variants, in which a single nucleotide change alters the codon to encode a different canonical AA (45) thereby generating canonical neoantigens (44). This is analogous to translational errors involving NCAAs, which generate non-canonical neoantigens. The antigenicity of a neoepitope can arise through several mechanisms: the presence of a non-encoded AA residue may create an anchor that increases the binding affinity of the peptide towards the MHC molecule, enhancing the presentation of neoepitopes against which the immune system is not tolerant (46) (Fig. 3). Alternatively, a non-coded AA residue that faces the T-cell receptor (TCR) binding site can alter TCR-binding properties, thereby enabling recognition by T-cells (45) (Fig. 3). AA substitutions can also influence protein processing and routing through MHC-loading compartments; for example, altered proteasomal processing may preserve ligands that would otherwise be degraded, thereby creating new neoepitopes (45). In addition, errors in translation can produce misfolded proteins exposing cryptic epitopes that are normally hidden in the native structure, but, when exposed, appear foreign to the immune system (46). Cross-reactivity to exogenous antigens and self-antigens, termed molecular mimicry, has been proposed as a mechanism of many autoimmune diseases. Exposure to a foreign antigen with a similar AA sequence or structure to host antigens can cause an autoimmune response (47). For example, there is evidence that antibodies induced against latent membrane protein one during Epstein-Barr virus infection might act as an inflammatory trigger by reacting with myelin basic protein (MBP) (48, 49). Autoreactivity against NCAA neoantigens could also occur through epitope mimicry, which requires either a significant overlap in the primary amino acid sequence or structural similarities between NCAA-containing and host epitopes (47).

## NCAA-containing protein neoepitopes and immunity

### Canavanine-induced systemic lupus erythematosus

Systemic lupus erythematosus (SLE) is a disorder characterized by the production of autoantibodies and chronic inflammation resulting from the deposition of the immune complexes in tissues (50). Epidemiological studies (51), including those involving monozygotic twins (52), suggest that both environmental factors and genetic predisposition contribute to the development of SLE. A potential link between the proteinogenic NCAA canavanine (2-amino-4-guanidinoxybutanoic acid) and a lupus-like syndrome was reported following the use of alfalfa (*Medicago sativa*) seeds to reduce plasma cholesterol levels (53, 54). Alfalfa seeds and sprouts contain around 2% canavanine by dry weight (55). Canavanine is a close structural analogue of the canonical AA arginine (Table 1) and acts as an antimetabolite in many biological systems (56, 57). *In vitro* studies have demonstrated that canavanine can replace up to 30% of arginine residues in proteins leading to apoptotic cell death (58). An SLE-like syndrome was also observed in monkeys fed alfalfa sprouts (59, 60) which led Malinow and colleagues to suggest that the immune system was reacting to “abnormal canavanyl proteins by giving rise to autoantibodies” (60). Re-exposure to canavanine reactivated the syndrome in monkeys in which an SLE-like syndrome had previously been induced (60). There were also reports of exacerbation of existing SLE in patients taking alfalfa tablets (58, 61). In a review article, Akaogi and colleagues supported the Malinow autoantibody hypothesis and proposed that the incorporation of canavanine into proteins could generate “immunogenic cryptic epitopes of self-proteins” (62). They reasoned that since aberrant misfolded proteins containing canavanine residues are ubiquitinated and undergo proteasomal degradation, the canavanyl-containing peptides could be presented on class MHC class I molecules (62). In addition, since L-canavanine induced apoptosis, the apoptotic cells containing canavanyl proteins also have the potential to be phagocytosed and processed by endosomal proteases to load canavanyl neo-epitope peptides on MHC class II molecules (62).

**Table 1:** Summary of the effects of proteinogenic NCAAs on the immune system

Canonical AA	Proteinogenic NCAA	NCAA source	Immune-effects	References
		Alfalfa (*Medicago sativa*)	SLE-like syndrome in humans. SLE-like syndrome in primates. Exacerbation of SLE in humans.	(53, 54) (59, 60) (58, 61)
		Sugar beets and fodder beets (*Beta vulgaris)*	Increased tumour immunogenicity and antitumour immune responses.	(20)
		Plants, oxidation of phenylalanine	Improved immune response in mice. Inhibition of hyperprogressive tumour in mice.	(73) (74)
		Cyanobacteria and diatoms	Innate immunity activation in neurons. Sensitisation of tumour cells in mice.	(85) (19)
		Plants, oxidation of tyrosine or phenylalanine	Increased in IgM and IgA levels in plasma. Thrombocytopenia with positive Coombs test. Positive antinuclear antibody test.	(87) (88, 89) (88)

Structures of the NCAAs and the 'parent' canonical amino acids are shown as well as the primary sources of the NCAAs in nature.

### Azetidine-2-carboxylic acid

Proline residues play a unique role in the protein backbone and are a primary determinant of protein tertiary architecture (63). Lubec and colleagues reported changes in the immunoreactivity of basement membrane collagen type IV in rat kidneys following oral administration of proteinogenic proline analogues (64, 65). Azetidine-2-carboxylic acid (A2C), a proteinogenic proline analogue from beets (*Beta vulgaris*) (Table 1), was shown to cause structural changes in both skin collagen (66) and hair (67). Rubenstein reasoned that since the displacement of proline by a NCAA might result in changes to the conformation and immunogenicity of a protein, A2C-containing neoepitopes generated on MBP could act as immunogenic triggers for multiple sclerosis (68). The hypothesis is supported by geographical and temporal links between higher incidences of multiple sclerosis and human exposure to A2C associated with beet cultivation for sugar production and its use as dairy cattle fodder (reviewed in (69)).

The most significant breakthrough in demonstrating that NCAA-containing neoepitopes were antigenic was made by Li and colleagues, who took advantage of the accelerated protein synthesis in tumor cells to increase the generation of aberrant A2C-containing proteins (20). When A2C was delivered to tumor tissue (4T1 tumor allografts) using a liposome nanoparticle-based delivery system, the regional generation of mistranslated proteins increased tumor immunogenicity and promoted antitumor immune responses, significantly prolonging the survival of the tumor-bearing mice (20). There was an increase in splenic CD8^+^ T cells, suggesting an MHC I immune response and an increase in CD80^+^ and CD86^+^ dendritic cells, which provide signals for T cell activation (20).

### Meta-tyrosine increases immune response

*Meta*-tyrosine is an allelochemical released into the soil by fescue grasses *(Festuca* spp.*)* to inhibit root growth in neighboring plants (29). It is also incorporated into proteins by mammalian cells (36, 70) and has potent anti-metastatic effects *in vivo* (71). *Meta*-tyrosine is a product of phenylalanine oxidation (Table 1) and is found in both free and protein-bound forms *in vivo* (reviewed in (72)). When administered to mice treated with lipopolysaccharides (LPS), *meta*-tyrosine prevented immunosuppression and restored the humoral immune response (73). Its presence increased the number of splenic CD4^+^ and CD8^+^ T-cells in the LPS-treated mice and also increased the expression of programmed death-ligand 1, a key marker of immunosuppression (73). The effect on both CD4^+^ and CD8^+^ T-cells indicates activation of both MHC class I and II pathways. Treatment with *meta*-tyrosine also inhibited metastases in mice bearing highly metastatic tumors (73). Furthermore, *meta*-tyrosine could enhance the overall immune response without producing a direct antitumor effect, indicating that immunogenicity can occur at a lower level of exposure than that required to produce cytotoxic effects (74).

### The neurotoxin β-methylaminoalanine (BMAA)

The cyanobacterial NCAA α-amino-β-methylaminopropionic acid, commonly known as β-methylaminoalanine (BMAA), attracted attention in the late 1960s due to its association with a complex neurological disorder among the residents of the South Pacific island of Guam (reviewed in (75)). Many *in vitro* and *in vivo* studies, including those involving primates, have supported the neurotoxic/excitotoxic properties of BMAA (75, 76). BMAA exists in both free and ‘protein-associated’ forms (77, 78); however, the nature of its association with proteins has been the subject of considerable debate. Based primarily on *in vitro* competition studies using radiolabeled L-BMAA, we proposed, that L-BMAA replaces L-serine during protein synthesis (79) (Table 1). Consistent with this observation, L-serine was later shown to protect against BMAA toxicity *in vitro* and *in vivo* (80, 81, 82). The BMAA-serine exchange was questioned by others who were unable to detect BMAA in proteins (83). Indeed, studies using isolated human tRNA synthetases suggested that BMAA is a substrate for human alanyl-tRNA synthetase but not human seryl-tRNA synthetase (84). In murine cortical neurons, BMAA was found to activated innate immunity, increasing toll-like receptor expression and promoting the production of chemokines and cytokines that are known mediators of innate immunity (85).

A lack of tumour-specific antigenic proteins, as seen in certain colorectal cancers, is a challenge for immunotherapy, and to address this, Tian and colleagues tested the ability of BMAA to artificially introduce neoantigens in a mouse colorectal carcinoma model (19). Treatment with BMAA inhibited tumor growth and activated dendritic cells (CD80^+^ and CD86^+^) as well as CD8^+^ T cell responses (19). In these studies, BMAA was shown to be misincorporated into proteins both *in vitro* and *in vivo*, specifically at serine sites (19). Despite a low level of incorporation, BMAA treatment significantly reduced tumor size and exhibited low systemic toxicity (19). The authors suggested that the generation of BMAA-containing neoepitopes could be a valuable approach to breaking immune tolerance without disrupting the systemic immune balance (19).

### L-3,4-dihydroxyphenylalanine (L-DOPA)

L-DOPA (Levodopa), a close structural analogue of L-tyrosine (Table 1), is the direct metabolic precursor of the neurotransmitter dopamine and has been used for more than 50 years to correct the dopamine deficiency resulting from dopaminergic neuron loss in Parkinson’s disease (PD). PD patients can be exposed to relatively high levels of this NCAA, and elevated levels of DOPA-containing proteins have been reported in post-mortem PD brains (86). Treatment with levodopa was reported to increase the synthesis of the cytokine interleukin-1 and increase IgM and IgA levels in the plasma of patients, leading the authors to suggest that levodopa had a selective action on cells of the immune system (87). In addition, there were several case reports of abnormally low platelets (thrombocytopenia) associated with L-DOPA therapy with positive reactions for antibodies against red blood cells (Coombs test) (88, 89) or an increased incidence of positive reactions for the antinuclear antibody test (88). Generally, an immune response would appear to be a rare occurrence in DOPA-treated patients, but it would be interesting to determine if the few patients who experience an immune reaction to DOPA would benefit from tyrosine or supplementation to prevent or reduce its incorporation into proteins.

## Conclusion

In this review, we present evidence that proteinogenic NCAAs can stimulate some level of immune response. This conclusion is based primarily on observations from animal studies and reports following human exposure, and not from targeted immunological studies. The principle, however, has now been effectively used to enhance tumor immunogenicity and prolong animal survival in murine cancer models (19, 20). The development of immune checkpoint inhibitors (ICIs) represented a major advancement in immunotherapy by preventing cancer cells from evading immune surveillance (90). There is considerable potential for proteinogenic NCAAs to be used in combination with ICIs to enhance tumor antigenicity, particularly in cancers with low mutational burdens, which are largely refractory to immunotherapy (91). Chemotherapy can also be used to increase neoantigens by inducing tumor gene mutations but can lead to irreversible gene mutations in normal cells and bone marrow suppression (92). Personalized tumor vaccines designed from whole-exome sequencing and MHC affinity prediction are expensive (93) and often limited in effectiveness, due to reliance on a single tumor antigen (94) whereas protein mistranslation induced by NCAAs offers a means of simultaneously generating multiple tumor neoantigens. It remains unclear, however, which NCAAs will provide the most effective therapeutic outcomes.

The higher rate of protein synthesis in tumors will increase the rate of neoantigen production *in situ* in response to NCAAs, while systemic effects can be reduced by providing sufficient levels of the parent amino acid to outcompete the NCAA for the tRNA synthetase (Fig. 2). Short term or intermittent treatment could potentially be employed to minimize unwanted effects from NPAA exposure. Targeting NCAAs to solid tumors, as used in the murine studies (19, 20), would also minimize damage to other tissues, and topical application could be an option for a local effect on skin cancers.

Many questions remain unanswered regarding the properties of proteinogenic NCAAs that enable them to stimulate a strong immune response. Structural studies have shown that tumor-generated neoepitopes need only differ slightly from their wild-type counterparts to be immunogenic in patients (46). Approximately 10% of immunogenic epitopes are linear, consisting of short peptides formed by continuous residues in a protein sequence, while the remainder are conformational epitopes, composed of spatially proximal residues within the three-dimensional structure of the folded protein (95). Generally, polar groups, hydrophobic groups, aromatic residues and reactive side chains enhance binding and antigenicity, but the ability to predict immunogenicity using computational methods has had limited success, particularly for conformational epitopes (96).

*Meta*-tyrosine induced a systemic immune response without any observable effects on tumor growth in a mouse model (74) suggesting that a low level of environmental exposure to proteinogenic NCAAs could stimulate a host immune response without the appearance of other toxic effects. A2C, found in fodder beets and sugar beet pulp, which are commonly used as winter fodder for dairy cattle in colder countries, has been implicated as an immune trigger for MS. Three independent studies have reported a strong correlation between the incidence of MS and cow’s milk consumption (97, 98, 99). Given that body growth, milk intake, and myelination are all at their highest levels during infancy, modification of MBP by substituting proline with A2C from consumed milk could embed immunogenic epitopes that trigger an immune response against MBP later in life (69). This aligns with observations linking MS risk to the place of residence in childhood (100).

We are at an early stage in our understanding of the immune response to neoepitopes generated by proteinogenic NCAAs, but this is an important emerging field with significant implications for cancer treatment and for understanding potential health risks associated with incidental environmental exposure to NCAAs. Further research is required to elucidate the mechanisms underlying these immune responses, identify which NCAAs are most likely to influence tumor antigenicity, and determine their safety profiles. Preclinical studies will be essential to optimize dosing strategies and evaluate toxicity, thereby informing future clinical applications.

## Data availability

All data generated or analyzed during this study are included in this published article and its supporting information files. Raw data is available upon request.

## Conflict of interest

The authors declare that they do not have any conflicts of interest with the content of this article.
